# Pattern of internet use for pregnancy-related information and its predictors among women visiting primary healthcare in Qatar: a cross-sectional study

**DOI:** 10.1186/s12884-021-04227-0

**Published:** 2021-11-05

**Authors:** Ayman Al-Dahshan, Mohamad Chehab, Ayatullah Mohamed, Noora Al-Kubaisi, Nagah Selim

**Affiliations:** 1 Department of Medical Education, Community Medicine Residency Program, Hamad Medical Corporation, Doha, Qatar; 2 Department of Clinical Affairs, Primary Health Care Corporation, Doha, Qatar; 3 Department of Public Health and Preventive Medicine, Faculty of Medicine Cairo University, Cairo, Egypt; 4 Department of Workforce Training, Primary Health Care Corporation, Doha, Qatar

**Keywords:** Pregnancy, Internet, Antenatal, Qatar

## Abstract

**Background:**

Internet usage has been steadily increasing and the available online information for pregnant women today is immense.

**Objective:**

This study aimed to investigate the prevalence of women who search for information relating to pregnancy on the internet and to identify predictors of this behavior among expecting women visiting primary health care (PHC) centers in Qatar.

**Methods:**

A cross-sectional study was conducted at the antenatal clinics of six PHC centers in Qatar from June 1 to December 31, 2019. Pregnant women were recruited through a systematic random sampling technique. Participants were interviewed using a structured questionnaire. Descriptive and analytic statistics were used when appropriate. A multivariable logistic regression analysis was constructed to identify the predictors of internet use for pregnancy-related information.

**Results:**

The study included 403 pregnant women (response rate: 87.9%). Most of them were in the 26–35 years age category (71.5%), in the second trimester (55.5%), and had 1–2 children at home (51.5%). The internet was the most common source (81.1%) of pregnancy-related information. The access to online information was mainly through websites (67.3%), mobile applications (48.3%), and social media platforms (39.7%). The most frequently searched topics online were fetal development (74.3%), diet during pregnancy (53.6%), and management of health problems (39.6%). The multivariable regression model identified the age group 26–35 years (AOR: 4.93; 95% CI: 2.14, 11.38), Arabs (AOR: 4.12; 95% CI: 2.03, 8.36), tertiary education (AOR: 5.22; 95% CI: 1.78, 15.33), being a housewife (AOR: 6.37; 95% CI: 1.44, 28.19), or employed (AOR: 5.56 95% CI: 1.31, 23.63), and having up to 2 children as independent predictors of internet use for pregnancy-related information.

**Conclusion:**

The internet was a commonly used source of health information among pregnant women in Qatar. Internet use was significantly associated with the age group of 26–35 years, Arabs, higher level of education, being employed or a housewife, and having up to two children.

## Introduction

Pregnancy is a transformative period in a woman’s life. As a result, pregnant women will seek health information to attain a sense of security in this regard. This information can be obtained from several sources such as health care professionals, family, friends, books, childbirth education classes, information centers, and the internet [1].

The internet has emerged as a convenient access portal for expecting women to have a vast amount of information pertaining to their pregnancy and childbirth [2]. The prevalence of internet use for health information during pregnancy varies across countries and is generally higher in developed nations than developing ones [2–9]. For example, a study among 1347 pregnant women in seven Italian cities found that the majority (86%) surfed the web for pregnancy-related information [3]. Another study of 335 Chinese pregnant women revealed that most (88.7%) have used the internet for health information [4]. On the other hand, less than half (45%) of 185 Turkish pregnant women employed the internet as a source of information on pregnancy [5]. Such variation could be attributable to several factors. One study found that young age, higher education, employment, and first pregnancy were predictors of internet use for gestation-related information [5]. The geographic origin of participants was an additional factor associated with internet use in another study [3].

Several reasons compel pregnant women to seek online health information such as the insufficiency of information given by health care providers and the ability to ask questions anonymously [8, 10]. Also, the internet has proven to be a platform for social support and sharing experiences [6, 11]. Likewise, pregnant women surf the web to gain knowledge about a wide variety of topics such as fetal development, pregnancy complications, delivery, and infant care. Other commonly searched themes include nutritional needs, physical exercise, and sexuality during pregnancy [2–5, 7].

In Qatar, pregnant women access antenatal care through specialized clinics across 25 primary healthcare (PHC) centers. The antenatal care package consists of clinical assessment, screening, management, and health promotion. During their prenatal period, women are followed up by primary care physicians and midwives over about eight clinical consultations [12]. Considering the internet’s popularity amongst the population worldwide and in Qatar [13], there is a need to explore if the internet is used to retrieve health information among expectant women and how they use it. This will help healthcare professionals in guiding and providing pregnant women with credible and high-quality online health sources. There is no previous study in Qatar that assessed the use of the internet as a source of information by women during pregnancy. Thus, the present study aimed to investigate the prevalence of women who search for information relating to pregnancy on the internet and to identify predictors of this behavior among expecting women visiting PHC centers. We hypothesized that the prevalence of internet use would be high among young and women of Arab nationality.

## Methods

### Study design and setting

This was an analytical cross-sectional study. It was conducted at the antenatal clinics (ANC) of PHC centers between June and December 2019. PHC centers are the most common first-line contact between community individuals and Qatar’s healthcare system. Each PHC center has a well-defined catchment population of different ethnic, cultural, social, and educational backgrounds; which offers a good representation of the community. The antenatal care of uncomplicated pregnancies is mainly provided through the primary healthcare system and is concluded by a referral to the nearest obstetric hospital at the 34th week of gestation [12].

### Study population and sampling

The target population included pregnant women, in any trimester, who speak Arabic or English and are attending the ANCs at the selected PHC centers ‎during the study period. Arabic and English are the most used languages in Qatar. The study excluded pregnant women who were unable to read or write. At the time of the study, there were 25 PHC centers distributed across the country’s three geographical regions (North, West, and Central). The present study was conducted across six PHC centres, where two health centers were chosen randomly from each region.

The recruitment of the participants was done through a systematic random sampling technique without replacement. The daily appointment list in each PHC center includes about 10 pregnant women, thus, we aimed to recruit 5 pregnant women per day (sampling fraction: 5/10). The ANC’s in-charge nurse generated the sampling frame from the daily appointment list in each chosen health center. The first participant of the day was selected from the first two participants randomly and subsequent participants were selected based on a fixed interval of “every other woman”.

### Sample size

The calculated sample size was 385 subjects based on a 5% absolute precision, 95% confidence, and a hypothesis that 50% of pregnant women were using the internet during pregnancy.

### Data collection

The data was collected through a self-administered questionnaire (described below). The pregnant women were approached by trained data collectors (midwives and nurses) at the waiting areas of the ANC’s in each selected PHC center. All potential participants were oriented about the study and assured that their participation was voluntary and had no effect on their quality of care. If they agreed to participate, they were requested to sign an informed consent and given a questionnaire in their preferred language (Arabic or English). Upon completion, the participants were appreciated for their participation and encouraged to ask any relevant questions. There was no incentive offered for the completion of the questionnaire.

### Questionnaire

A structured and self-administered questionnaire was developed by the authors through an extensive review of the literature. It achieved face and content validity through critical review by an expert panel of community medicine consultants and experts in the field. In addition, test-retest reliability was assessed (coefficients = 0.9). It encompassed three main sections and seventeen close-ended questions. Section A consisted of five questions on the socio-demographic characteristics. Section B included seven questions related to the characteristics of the current pregnancy and the sources of information. Section C contained five questions on the patterns of internet use for pregnancy-related information. The questionnaire was translated and back-translated (English-Arabic) by two independent translators and any aberrancy was corrected. After which, it was piloted on twenty pregnant women to assess its comprehensiveness and clarity. No modifications were made after the pilot phase.

### Statistical analysis

The data were analyzed using IBM SPSS Statistics for Windows, version 23.0 (IBM Corp., Armonk, N.Y., USA). Descriptive statistics were calculated for continuous and categorical variables. Pearson’s chi-squared test was used to assess the association between internet use and the independent variables. A multivariable logistic regression analysis was constructed to identify the independent predictors of internet use for pregnancy-related information. All variables with *p*-values < 0.25 in univariable analysis were included in the multivariable analysis. We performed the Hosmer-Lemeshow goodness of fit test and its findings indicate that our model adequately fits the data (*p*-value = 0.561). The adjusted odds ratio (AOR) with their 95% confidence interval were reported for all predictors. The level of statistical significance was set at 0.05.

## Results

### Demographic characteristics of participants

A total of 403 out of 458 invited pregnant women participated in the study (response rate: 87.9%) with time constraint being the main reason for non-participation. The characteristics of the study participants are shown in Table 1. Most respondents (71.5%) were in the 26–35 years age category and had tertiary education (69.9%). Also, more than half of the participants were in the second trimester (55.5%) and had 1–2 children at home (51.5%). Most pregnant women reported having a health problem (77.4%) during the current pregnancy, such as heartburn (31.8%), morning sickness (25.8%), and low back pain (25.3%).

**Table 1:** Characteristics of participants and obstetric history (*N* = 403)

Variable	n (%)
Age (year)	
18–25	74 (18.5)
26–35	286 (71.5)
36 or more	40 (10.0)
Nationality	
Arab	220 (55.1)
Non-Arab^a^	179 (44.9)
Level of education	
Primary education	28 (7.0)
Secondary education	93 (23.1)
Tertiary education^b^	281 (69.9)
Occupation	
Housewife	242 (60.0)
Working	147 (36.5)
Student	14 (3.5)
Gravida	
Primigravida	107 (26.6)
Multigravida	296 (73.4)
Number of living children	
No children	121 (30.1)
1–2 children	208 (51.7)
3 children or more	73 (18.2)
Trimester	
First	25 (6.3)
Second	221 (55.5)
Third	152 (38.2)
Gender of the fetus	
Male	139 (34.5)
Female	104 (25.8)
Prefer not to say/I don't know	160 (39.7)
Health problems during the current pregnancy	
No	91 (22.6)
Yes^c^	312 (77.4)
Heartburn	128 (31.8)
Morning sickness	104 (25.8)
Low back pain	102 (25.3)
Gestational diabetes	87 (21.6)
Vomiting	70 (17.4)
Anemia	60 (14.9)
Others^d^	51 (12.7)

^a^ Non-Arab includes Asian, Western and African

^b^ College or university degree

^c^ Not mutually exclusive categories

^d^ Urinary tract infection, depression, hypertension

### Sources of pregnancy-related information

Table 2 shows the sources of pregnancy-related information among the study participants during their current pregnancy. The most frequently reported source was the internet (81.1%). Other popular sources for gestation-related information included healthcare providers (64.4%), family members (44.8%), and friends (24.9%). Only a small group (9.2%) of respondents had attended an educational activity on pregnancy and most (62.2%) did so at the PHC center.

**Table 2:** Sources of pregnancy-related information during the current pregnancy (*N* = 403)

Source of information	n (%)
Internet	
Yes	327 (81.1)
No	76 (18.9)
Healthcare providers	
Yes	259 (64.4)
No	143 (35.6)
Family members	
Yes	188 (44.8)
No	222 (55.2)
Friends	
Yes	100 (24.9)
No	302 (75.1)
Books or magazine	
Yes	39 (9.7)
No	363 (90.3)
Television	
Yes	20 (5.0)
No	382 (95.0)
Participation in educational activity	
Yes	37 (9.2)
No	366 (90.8)
*Place of the educational activity (n = 37)*	
Primary healthcare center	23 (62.2)
Governmental hospital	6 (16.2)
Private antenatal clinic	5 (13.5)
Private hospital	3 (8.1)

### Internet use for pregnancy-related information

Table 3 shows the pattern of internet use for pregnancy-related information (*N* = 327). Many pregnant women (42.9%) reported using the internet daily and more than a quarter (27.9%) used it 3–4 times a week. Access to online information was mainly through websites (67.3%), mobile applications (48.3%), and social media platforms (39.7%). Moreover, most participants (89.2%) attributed their internet usage to the need for more pregnancy-related information. On the other hand, more than a quarter (28%) of internet users sought to share their experiences with others online.

**Table 3:** Pattern of internet use for pregnancy-related information among study participants (*N* = 327)

Variable	n (%)
Frequency of internet use during the current pregnancy	
Everyday	140 (42.9)
3–4 times a week	91 (27.9)
1–2 times a month	49 (15.0)
Few times	46 (14.1)
Methods used to access pregnancy-related information	
Website	218 (67.3)
Mobile application	157 (48.3)
Social media	129 (39.7)
Forum	27 (8.3)
Main purpose of searching the internet during pregnancy	
To look for pregnancy-related information	290 (89.2)
To share the experience with others	91 (28.0)
To look for support	27 (8.3)
To talk anonymously on sensitive issues	21 (6.5)

**Fig. 1:**
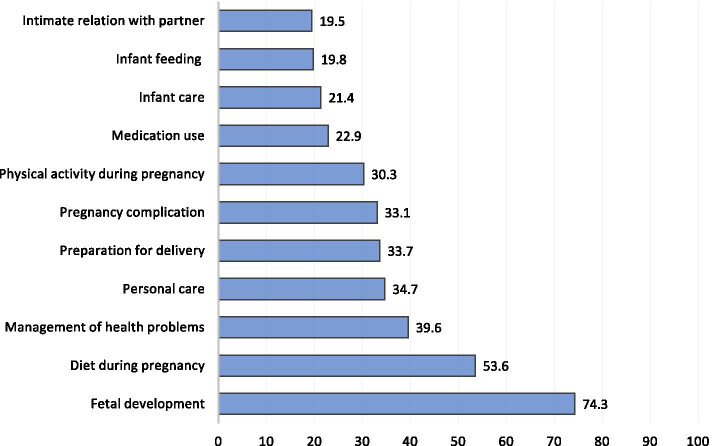
Main topics searched online by study participants (*N* = 327)

Figure 1 shows the main topics searched online, the most frequently reported themes were fetal development (74.3%), diet during pregnancy (53.6%), management of health problems (39.6%), personal care (34.7%), and preparation for delivery (33.7%). Infant care (21.4%), infant feeding (19.8%), and intimacy (19.5%) were the least frequently reported topics among pregnant women.

When the relationship between internet use and the participants’ characteristics was examined using the chi-squared test, a statistically significant difference was observed between internet use and the age group (*p* < 0.001), level of education (*p* < 0.001), occupation (*p* = 0.003), number of living children (*p* = 0.02), and stage of pregnancy (*p* = 0.009). It was determined that pregnant women between the ages of 26 and 35 years, with a tertiary education, currently employed, having 1–2 children at home, and in the last stage of pregnancy (third trimester) were more likely to access the internet for information on pregnancy (Table 4).

**Table 4:** The relationship between participants’ characteristics and the use of internet for pregnancy-related information (*N* = 403**)**

Variable	Internet use for pregnancy-related info		*p*-value
	Yes, n (%)	No, n (%)	
Age (year)			< 0.001*
18–25	48 (64.9)	26 (35.1)	
26–35	246 (86.0)	40 (14.0)	
36 or more	30 (75.0)	10 (25.0)	
Nationality			0.168
Arab	184 (83.6)	36 (16.4)	
Non-Arab	140 (78.2)	39 (21.8)	
Level of education			< 0.001*
Primary education	17 (60.7)	11 (39.3)	
Secondary education	60 (64.5)	33 (35.5)	
Tertiary education	249 (88.6)	32 (11.4)	
Occupation			0.003*
Housewife	189 (78.1)	53 (21.9)	
Working	130 (88.4)	17 (11.6)	
Student	8 (57.1)	6 (42.9)	
Gravida			0.734
Primigravida	88 (82.2)	19 (17.8)	
Multigravida	239 (80.7)	57 (19.3)	
Number of living children			0.024*
No children	100 (82.6)	21 (17.4)	
1–2 children	175 (84.1)	33 (15.9)	
3 children or more	51 (69.9)	22 (30.1)	
Trimester			0.009*
First	18 (72.0)	7 (28.0)	
Second	171 (77.4)	50 (22.6)	
Third	135(88.8)	17 (11.2)	
Gender of the fetus			0.074
Male	121 (87.1)	18 (12.9)	
Female	83 (79.8)	21 (20.2)	
Prefer not to say/I don’t know	123 (76.9)	37 (23.1)	
Presence of health problems during the current pregnancy			0.039*
No	76 (74.5)	26 (25.5)	
One health problem	103 (79.2)	27 (20.8)	
Two or more health problems	148 (86.5)	23 (13.5)	

* Statistically significant (*p* < 0.05)

The multivariate logistic regression analysis is shown in Table 5. Participants aged 26–35 years were about five times more likely to use the internet for pregnancy-related information compared to those in the age group of 18-25 years (AOR: 4.93; 95% CI: 2.14, 11.38). In addition, the following variables were identified as independent predictors of internet use for pregnancy-related information: Arab nationality (AOR: 4.12; 95% CI: 2.03, 8.36), tertiary education (AOR: 5.22; 95% CI: 1.78, 15.33), being a housewife (AOR: 6.37; 95% CI: 1.44, 28.19), or employed (AOR: 5.56; 95% CI: 1.31, 23.63), and having no children or up to 2 children.

**Table 5:** Predictors of internet use for pregnancy-related information (*N* = 403)

Variable	Adjusted OR (95% CI)	*p-value*
Age (year)		
18–25	Reference	
26–35	4.93 (2.14, 11.38)	< 0.001*
36 or more	2.84 (0.87, 9.28)	0.084
Nationality		
Arab	4.12 (2.03, 8.36)	< 0.001*
Non-Arab	Reference	
Level of education		
Primary education	Reference	
Secondary education	1.32 (0.47, 3.71)	0.596
Tertiary education	5.22 (1.78, 15.33)	0.003*
Occupation		
Housewife	6.37 (1.44, 28.19)	0.015*
Working	5.56 (1.31, 23.63)	0.020*
Student	Reference	
Number of living children		
No children	4.21 (1.51, 11.74)	0.006*
1–2 children	3.41 (1.45, 8.01)	0.005*
3 children or more	Reference	
Trimester		
First	Reference	
Second	0.77 (0.25, 2.37)	0.641
Third	2.21 (0.61, 7.98)	0.227
Gender of the fetus		
Male	1.82 (0.86, 3.82)	0.116
Female	0.91 (0.42, 1.97)	0.808
Prefer not to say/I don’t know	Reference	
Health problems during the current pregnancy		
No	Reference	
Yes	0.96 (0.48, 1.89)	0.896

*OR* Odds ratio, *CI* Confidence intervals; *Statistically significant (*p* < 0.05)

## Discussion

The current study investigated internet usage as a source of health information among pregnant women in Qatar. It was found that accessing the internet for such purpose was prevalent (81.1%) among our study participants and significantly associated with a specific age group, Arabs, higher level of education, being employed or a housewife, and having up to two children.

In this study, pregnant internet users represented a majority (88.1%) of the total sample. This result aligns with the findings of earlier studies that reported a high prevalence of internet use as a source of health information during pregnancy in developed countries such as Sweden, China, and Canada [2, 4, 7]. The widespread use of online sources for health information during pregnancy can be explained by increased internet availability among the global population. It is estimated that by the end of 2019, 4.1 billion individuals or more than half (53.6%) of the global population were using the internet. This represents almost a seven-fold increase in the percentage of internet users since 2001 at 8% [14]. Another driving force behind this trend could be the abundance of online health information. However, the credibility of this information varies widely among different sources and it becomes the responsibility of the consumer to navigate, seek, and validate it [15].

In addition to that, nearly two-thirds (64.4%) of participants recognized their healthcare providers as a source of pregnancy-related information during the current gestation. Subsequently, healthcare professionals in the country must adopt a patient-centered communication strategy through open dialogue about the use of the internet for health information. Also, pregnant women are a vulnerable cohort and must be empowered with the proper skills to access online health information and encouraged to discuss this information with their health care providers. Similarly, Qatar’s health officials must collaborate with experts in the field to develop comprehensive, user-friendly, and culturally appropriate online resources for pregnant women. In the meantime, health professionals must be trained on how to identify and recommend valid online sources for pregnant internet users [16].

Another study finding was the low level of participation in educational activities related to pregnancy, where only a small proportion (9.2%) of participants acknowledged doing so. Likewise, there is a declining trend of attending childbirth education classes as evidenced in the literature. Childbirth education classes have a well-documented positive impact on pregnancy outcomes [17–19]. Hence, organizers of such classes in the country need to integrate internet use as part of the class activities in an effort to attract this large cohort of pregnant women. The classes will offer an organized environment for the sharing and flow of credible pregnancy-related information without the overwhelming and puzzling aspect of the online environment. Given the COVID-19 pandemic, there is an opportunity to offer these classes as part of the virtual consultations taking place across Qatar’s PHC centers.

Regarding the most searched online topics, fetal development and diet during pregnancy were the most frequently reported themes. These results corroborate the evidence of an earlier systematic review that detected these two topics as the most common areas of interest among expecting mothers on the internet [20]. Seeking online information about fetal development might reflect a maternal need for comfort or reassurance in this regard. Many pregnant women will be concerned regarding their fetal wellbeing. Regarding nutrition during pregnancy, a study in China associated this concern with the popular underlying belief that nutrition plays an important role in maternal health [21]. On the other hand, intimacy was the least searched online theme in this study. This could be due to the sensitivity of such issues among the conservative community in Qatar. Moreover, healthcare providers should be aware of these themes and provide more evidence-based information to their pregnant women in a timely and comprehensible manner. Specifically, health professionals may want to focus on the normal intrauterine development of the fetus during the different stages of pregnancy as well as nutritional guidance to pregnant women during this critical phase of their life.

Our study has identified a significant association between several participant characteristics and using the internet for pregnancy-related information. First, participants between the ages of 26 and 35 years were the most (86%) likely to surf the internet for health information during pregnancy. This result conforms with that of a Turkish study in which participants aged 25–34 years reported using the internet more frequently than their younger (18–24 years) and older (≥ 35 years old) peers [5]. On the other hand, an earlier study among 193 pregnant Swedish women did not find any significant association between age and using the internet for health information [2]. This area requires further research to understand the drivers behind such differences.

Secondly, pregnant women with tertiary education as well as those who were employed reported the highest internet usage as a source of pregnancy-related information. Similarly, a study on the information needs and health-seeking practices of pregnant women found that the internet was not widely accessed by pregnant women with a low income and low education level [22]. However, unemployed participants in our study might still have a high income depending on their spouses’ or families’ financial status. As such, we cannot infer a similar explanation in this study. Nevertheless, being highly educated is associated with possessing the skills to search for health information online [20].

Third, our study detected that pregnant women, with up to 2 children at home, used the internet more frequently than their peers. Moreover, there was a minimal difference in internet use between those participants who had no children (82.6%) and those having 1–2 children at home (84.1%). These results are like that of a multicenter Italian study that found no significant difference regarding internet access among primiparous and multiparous women [6]. In contrast, a study among American pregnant women found that nulliparous participants (50.3%) were two times more likely to use the internet than their multiparous counterparts (21.3%) [23]. A similar finding was noted among pregnant women in our study, with 3 or more children at home, who reported the least internet use (69.9%) for online information. Thus, pregnant women with more than two children might have gained enough knowledge and experience from the earlier pregnancies. Subsequently, these participants have assimilated a higher level of confidence that explains their relative independence from using the internet as a source of pregnancy-related information.

Finally, the respondents who were in the third trimester of their pregnancy reported more usage of the internet as a source of health information (88.8%) in comparison to those in the first (72%) and second trimesters (77.4%). So, our participants portrayed an increasing need for health information throughout their pregnancy. In contrast, a study among Chinese pregnant women revealed a drastic decrease in internet usage from the first (81.5%) till the third trimester (5.1%). This was attributed to a stronger need for information during the early stage of pregnancy, where the clinical consultations under the Chinese antenatal program begin from the 20th week of gestation [4]. On the other hand, the Qatari antenatal program initiates clinical visits early on from the 6th week of pregnancy and offers up to six visits during the first trimester [24]. Consequently, more research is needed to understand the increasing trend of internet use by stage of pregnancy among pregnant women in Qatar.

The current study has some limitations. First, it was conducted among pregnant women attending the antenatal clinics under the Primary Health Care Corporation, Qatar’s governmental primary health care provider. So, our findings cannot be extrapolated onto the pregnant women seeking antenatal care in the private health sector. Another limitation of this study was that certain questions in the questionnaire relied on pregnant women’s ability to remember. Additionally, the cross-sectional study design cannot generate enough evidence to establish causality.

Nevertheless, our study has several strengths. It is the first study to assess the use of the internet as a source of pregnancy-related information among pregnant women in Qatar. Moreover, the high level of response among the participants and the completeness of the questionnaires can be attributable to employing nurses and midwives for administering the questionnaire. Furthermore, a probability sampling technique was employed to select participants from governmental PHC center which cater to a population of diverse backgrounds. Therefore, the results may be generalizable to most pregnant women attending primary care in Qatar. In addition, the study has identified several gap areas that can be used in enhancing the quality and comprehensiveness of antenatal care in the country.

## Conclusion

In conclusion, the present study showed that the internet was a widely used source of health information among pregnant women in Qatar. Internet use was significantly associated with the age group of 26–35 years, Arabs, higher level of education, being employed or a housewife, and having up to two children. These findings will inform public health officials on guiding pregnant women to high-quality and valid online health sources.

## Data Availability

The datasets used and/or analysed during the current study are available from the corresponding author on reasonable request.
